# Breaking down the recovery of O_2_ pathway: Peripheral extraction recovery pattern defines exercise capacity and clinical outcomes

**DOI:** 10.14814/phy2.70337

**Published:** 2025-04-13

**Authors:** Ahmed El Shaer, Mariana Garcia‐Arango, Anas Abed, Shannon Heffernan, Yuning Wang, Tashfeen Javed, Amirhossein Esmaeeli, Abdul Wahab Arif, Ran Tao, Naga Dharmavaram, James Runo, Jill N. Barnes, Farhan Raza

**Affiliations:** ^1^ Department of Internal Medicine, School of Medicine and Public Health University of Wisconsin‐Madison Madison Wisconsin USA; ^2^ Division of Cardiovascular Medicine, School of Medicine and Public Health University of Wisconsin‐Madison Madison Wisconsin USA; ^3^ Department of Statistics, School of Computer, Data & Information University of Wisconsin‐Madison Madison Wisconsin USA; ^4^ Fatima Jinnah Medical University Lahore Pakistan; ^5^ Division of Pulmonary and Critical Care, School of Medicine and Public Health University of Wisconsin‐Madison Madison Wisconsin USA; ^6^ Department of Kinesiology University of Wisconsin‐Madison Madison Wisconsin USA

**Keywords:** heart failure with preserved ejection fraction, heart rate recovery, peak O_2_, peripheral extraction recovery, pre‐capillary pulmonary hypertension, V̇O_2_ recovery

## Abstract

Poor recovery pattern of oxygen consumption (V̇O_2_) post‐exercise is associated with adverse clinical outcomes. However, it remains unknown which component of the O_2_ pathway (Fick principle) defines this prognostic risk, for example, peripheral extraction, stroke volume, heart rate. Retrospective cohort study included 120 participants (heart failure with preserved ejection fraction: HFpEF = 68, pre‐capillary pulmonary hypertensio *n* = 31, non‐cardiac dyspnea = 21). Percent recovery metrics were calculated as the percent reduction of each hemodynamic variable from peak exercise to recovery, for example, (exercise‐recovery)/exercise ×100%. Overall, the mean age (standard deviation) was 62.6 (14.4) years and 54% were females. Among the three groups (HFpEF, pre‐capillary pulmonary hypertension, non‐cardiac dyspnea), recovery patterns of O_2_ pathway components were statistically non‐significant. Peripheral extraction recovery (*r* ^2^ = 0.43, *p* < 0.001) and heart rate recovery (*r* ^2^ = 0.25, *p* < 0.001) correlated with peak V̇O_2_, but only peripheral extraction recovery remained significant in multivariate analysis (*p* = 0.01). Peripheral extraction recovery (<41%; median) demonstrated poor one‐year survival from mortality and heart failure hospitalizations (HR 2.82; CI 95% 1.38–5.74, *p* = 0.003). Peripheral extraction recovery pattern is the most significant component of the O_2_ pathway and defines adverse outcomes. Physiologically, it elucidates the importance of skeletal muscle and peripheral vascular function.

## 1 INTRODUCTION

Peak oxygen consumption (V̇O_2_) is a unifying prognostic factor among different pulmonary hypertension (PH) phenotypes, such as heart failure with preserved ejection fraction (HFpEF) and pre‐capillary PH (Humbert et al., 2023). To quantify multi‐system limitations to exercise tolerance and O_2_ delivery pathway, cardiopulmonary exercise testing (CPET) is crucial for identifying limitations in cardiac, pulmonary, or peripheral muscle function; each necessitating different treatments (Raza et al., 2022). In addition to cardiac and pulmonary vascular limitations, peripheral extraction abnormalities (indicating microcirculation and skeletal muscle health) can be a predominant factor in poor O_2_ delivery and uptake during exercise (Dhakal et al., 2015; Kitzman et al., 2014).

Beyond the prognostic value of V̇O_2_ achieved with peak exercise, the rapid recovery of V̇O_2_ and heart rate (HR) post‐exercise has been linked to good cardiovascular health (Bailey et al., 2018; Qiu et al., 2017) and abnormal recovery patterns (e.g., prolonged times) for V̇O_2_ after exercise are associated with adverse clinical outcomes (Chatterjee et al., 2013). However, it remains unknown which components of the O_2_ pathway (e.g., stroke volume, heart rate, peripheral extraction) define the physiological significance and clinical relevance of V̇O_2_ recovery. We hypothesize that in comparison to stroke volume and heart rate recovery, the peripheral extraction recovery pattern has stronger correlations with exercise capacity and clinical outcomes among different PH phenotypes, and hence, is the biggest component that defines the physiological and clinical relevance of O_2_ pathway recovery. The goal of the current study is to elucidate the post‐exercise recovery patterns of different components of the O_2_ pathway among different PH phenotypes. Additionally, we aim to characterize the prognostic relevance of these recovery patterns in defining correlations with exercise capacity and clinical adverse outcomes.

## 2 METHODS

### 2.1 Study design and population

This retrospective cohort study included participants age >18 years with exercise intolerance and New York Heart Association Class II–III who presented to the PH clinic at the University of Wisconsin‐Madison. All participants had an echocardiogram and a clinically indicated exercise invasive hemodynamic study. Exclusionary criteria included LVEF <50%, supplemental oxygen use, serum creatinine >2 mg/dL or on dialysis, primary lung diseases with forced vital capacity <60% predicted, and severe valvular heart disease. All participants underwent rest‐to‐exercise cardiopulmonary exercise testing using a semi‐recumbent ergometer with simultaneous right heart catheterization and expired gas analysis. In addition to right heart catheterization metrics, key gas exchange variables included peak V̇O_2_ and V_E_/VCO_2_ slope (minute ventilation/carbon dioxide production), as previously described (Raza et al., 2022; Tao et al., 2024). They were categorized into three groups based on their rest and exercise hemodynamics, following the 2022 ESC/ERS guidelines (Humbert et al., 2023).

### 2.2 Statistical analysis

Continuous variables were reported as means ± standard deviation (SD) and medians (interquartile range). Categorical variables were reported as frequencies and percentages. The Wilk–Shapiro test assessed the normality of continuous variables. For two‐group comparisons, Student's *t*‐test or Mann–Whitney test was used. For three‐group comparisons, ANOVA or Kruskal‐Wallis test with post hoc adjustments was used. Categorical variables were compared using chi‐square or Fisher exact test. Univariate linear regression evaluated predictors of exercise capacity (peak V̇O_2_) with fixed categorical variables (HFpEF, Pre‐capillary PH, NCD). Model selection for multivariate regression used the Akaike Information Criterion (AIC). Survival analyses utilized Kaplan–Meier with log‐rank test and Cox regression. Analyses were performed with R studio (version 4.2.2) and GraphPad Prism (version 10.0.0).

## 3 RESULTS

### 3.1 Baseline characteristics and echocardiographic parameters

Baseline characteristics and echocardiographic parameters for HFpEF (*n* = 68), pre‐capillary PH (*n* = 31), and NCD (*n* = 21) are summarized in Table 1. As expected, HFpEF participants had a higher body mass index and more frequent comorbidities, with a reduced six‐minute walk test distance as compared to the pre‐capillary PH and NCD groups. Regarding echocardiographic parameters, both HFpEF and the pre‐capillary PH groups had reduced right ventricular‐vascular coupling to a similar level (TAPSE/PASP = 0.43 ± 0.19 mm/mmHg and 0.42 ± 0.17 mm/mmHg respectively).

**TABLE 1:** Baseline and hemodynamic characteristics of the three cohorts.

	HFpEF	Precapillary PH	NCD	*p*‐Value
	(*n* = 68)	(*n* = 31)	(*n* = 21)	
Age, years	64.9 ± 13.4	62.7 ± 12.9	54.6 ± 16.6	0.01*
Female sex, *n* (%)	32 (47%)	18 (58%)	15 (71%)	0.13
Body Mass Index, kg/m^2^	33.8 ± 8.5	27.1 ± 5.7	28.3 ± 4.9	<0.001*, ^†^
NYHA functional class, median (IQR)	3 (1)	2 (1)	2 (1)	0.04
6‐min walk distance, m	266.6 ± 96.6	355.9 ± 111.2	430.9 ± 66.7	<0.001*, ^†^
Comorbidities				
Hypertension, *n* (%)	58 (85%)	18 (58%)	9 (43%)	<0.001*, ^†^
Diabetes mellitus, *n* (%)	21 (31%)	4 (13%)	1 (5%)	0.01*
COPD, *n* (%)	11 (16%)	1 (3%)	3 (14%)	0.19
Coronary artery disease, *n* (%)	27 (40%)	7 (23%)	0 (0%)	<0.001*, ^‡^
Atrial fibrillation, *n* (%)	31 (46%)	3 (10%)	4 (19%)	<0.001*, ^†^
Echocardiographic parameters				
LVEF, %	60.5 ± 6.0	61.8 ± 3.1	61.2 ± 5.4	0.58
LVMI, g/m^2^	97.7 ± 46.8	83.5 ± 54.4	69.1 ± 17.1	0.049*
Epicardial adipose tissue, mm	6.4 ± 2.6	5.1 ± 1.8	4.9 ± 3.0	0.02^†^
LAVI, mL/m^2^	40.4 ± 14.6	27.6 ± 15.8	29.8 ± 15.3	0.003^†^
TAPSE, mm	19.6 ± 6.1	18.2 ± 4.1	21.1 ± 4.9	0.23
PASP, mmHg	51.8 ± 21.9	50.2 ± 18.6	29.6 ± 8.7	0.001*, ^‡^
TAPSE/PASP ratio, mm/mmHg	0.43 ± 0.19	0.42 ± 0.17	0.74 ± 0.23	<0.001*, ^‡^
Average E/E' ratio	13.2 ± 8.2	12.1 ± 9.3	7.9 ± 3.1	0.01*
Eccentricity Index (systole)	1.1 ± 0.3	1.3 ± 1.1	0.9 ± 0.2	0.3
RV:LV ratio	1.0 ± 1.1	0.9 ± 0.2	0.8 ± 0.2	0.67
Rest hemodynamics				
Heart rate, bpm	69.3 ± 11.5	67.6 ± 8.6	77.0 ± 9.0	0.004*, ^‡^
Fick CO, L/min	5.2 ± 1.4	4.7 ± 1.2	5.7 ± 0.9	0.01^‡^
V̇O_2_ (mL/min)	278 ± 57	241 ± 47	254 ± 44	0.01^†^
Peripheral extraction, mL/dL	5.8 ± 1.4	5.6 ± 1.2	4.6 ± 0.7	0.002*, ^‡^
Stroke volume index, mL/m^2^	36.8 ± 9.3	37.5 ± 8.7	39.9 ± 6.7	0.37
Peak exercise hemodynamics				
Heart rate, bpm	93.5 ± 22.8	103.5 ± 19.6	124.6 ± 21.8	<0.001*, ^‡^
Fick CO, L/min	8.8 ± 3.3	8.7 ± 2.4	12.3 ± 3.1	<0.001*, ^‡^
V̇O_2_ (mL/min)	924 ± 328	888 ± 308	1238 ± 468	0.02*, ^‡^
Peripheral extraction, mL/dL	11.1 ± 2.8	10.5 ± 2.7	9.7 ± 2.2	0.17
Stroke volume index, mL/m^2^	43.8 ± 11.9	43.1 ± 10.9	52.6 ± 9.8	0.01*, ^‡^
mPAP/CO slope, mmHg/L.min^−1^	7.1 ± 5.6	5.4 ± 2.5	2.1 ± 0.8	<0.001*, ^‡^
PAWP/CO slope, mmHg/L.min^−1^	5.8 ± 6.0	1.6 ± 0.9	1.1 ± 0.6	<0.001*, ^†^
Recovery hemodynamics				
Heart rate, bpm	78.0 ± 14.7	82.1 ± 15.7	95.9 ± 16.2	<0.001*
V̇O_2_ (mL/min)	411 ± 113	352 ± 69	373 ± 117	0.04^†^
Peripheral extraction, mL/dL	6.8 ± 2.1	6.0 ± 1.6	4.5 ± 1.0	<0.001*, ^‡^
Stroke volume index, mL/m^2^	41.0 ± 10.6	40.5 ± 10.9	48.4 ± 11.6	0.09
CPET				
Work, watts	61.2 ± 34.2	66.7 ± 32.3	103.5 ± 50.4	<0.001*, ^‡^
V̇O_2_, mL/kg/min	9.8 ± 3.2	11.5 ± 3.6	16.1 ± 6.5	<0.001*, ^‡^
V̇O_2_ predicted, %	47.8 ± 14.4	51.8 ± 17.8	68.6 ± 26.4	<0.001*, ^‡^
V_E_/V̇O_2_ slope	39.7 ± 8.9	42.8 ± 9.7	36.7 ± 6.8	0.048^‡^
Peak exercise RER	0.97 ± 0.11	1.01 ± 0.09	1.06 ± 0.14	0.01*
dCO/dV̇O_2_	5.2 ± 2.4	6.5 ± 2.3	7.1 ± 2.3	0.003*

Abbreviations: CO, cardiac output; COPD, chronic obstructive pulmonary disease; CPET, cardiopulmonary exercise testing; E/E', ratio of early diastolic mitral blood flow velocity to mitral annulus velocity; HFpEF, Heart failure with preserved ejection fraction; LAVI, left atrial volume index; LV, left ventricle; LVEF, left ventricular ejection fraction; LVMI, left ventricular mass index; mPAP, mean pulmonary arterial pressure; NCD, non‐cardiac dyspnea; NYHA, New York heart association; OSA, obstructive sleep apnea; PASP, pulmonary artery systolic pressure; PAWP, pulmonary artery wedge pressure; PH, pulmonary hypertension; RER, respiratory exchange ratio; RV, right ventricle; TAPSE, tricuspid annular pulmonary systolic excursion; V_E_/VCO_2_, minute ventilation/carbon dioxide production; V̇O_2_, oxygen consumption.

* *p* < 0.05 for comparison of HFpEF and NCD participants.

^†^ *p* < 0.05 for comparison of HFpEF and Pre‐capillary PH.

^‡^ *p* < 0.05 for comparison of Pre‐capillary PH and NCD participants.

### 3.2 Exercise hemodynamic and CPET parameters

Hemodynamic and CPET measurements performed at rest and during peak exercise are summarized in Table 1. The NCD cohort achieved a higher peak heart rate and cardiac output at peak exercise compared to the HFpEF and pre‐capillary PH groups. HFpEF and pre‐capillary PH groups had a similar exercise limitation based on peak workload (watts), peak V̇O_2_, and V_E_/VCO_2_ slope.

### 3.3 Recovery metrics of O_2_ pathway components: %V̇O_2_r, %PEr, %HRr, %SVr

**FIGURE 1:**
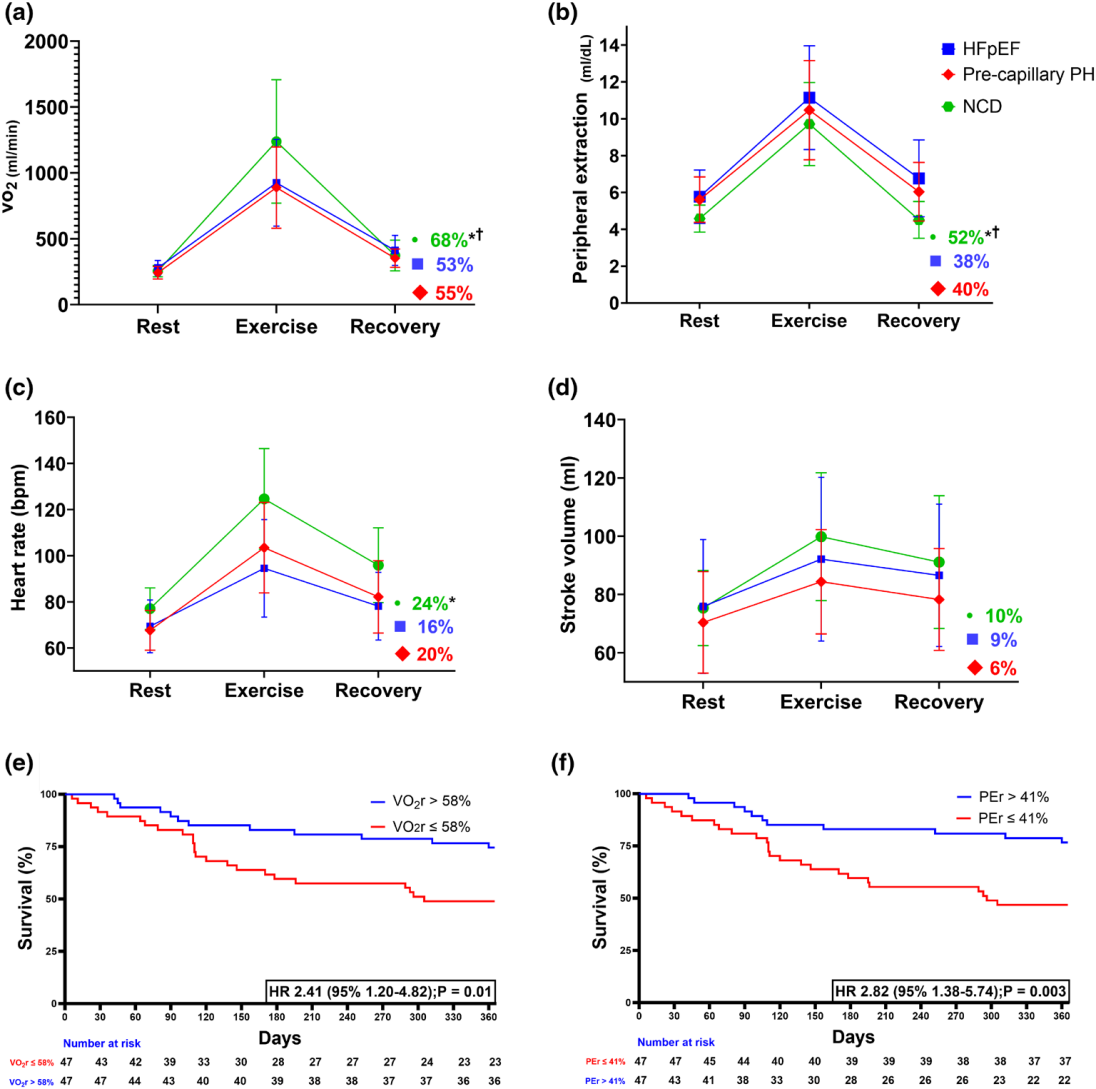
Percent reduction of O_2_ pathway components and impact on outcomes. Rest‐Exercise‐Recovery (2‐min) patterns and percent reduction of exercise‐to‐recovery of VO_2_ (a), peripheral extraction (b), heart rate (c), and stroke volume (d) among three hemodynamic groups of HFpEF, pre‐capillary PH, and NCD. Impact of median values of V̇O_2_r = 58% (e) and PEr = 41% (f) on composite outcome of Survival from mortality and heart failure hospitalization at one‐year follow‐up after the exercise hemodynamic study. For *p*‐value <0.05 between stages of “exercise” and “recovery” (a–d): *HFpEF versus NCD. ^†^Pre‐capillary PH versus NCD. *n* = 94 due to lack of follow‐up of 26 participants. FpEF, heart failure with preserved ejection fraction; NCD, non‐cardiac dyspnea; PEr, peripheral extraction percent reduction; PH, pulmonary hypertension; V̇O_2_, oxygen consumption; V̇O_2_r, V̇O_2_ percent reduction.

Recovery metrics were recorded 2 min after exercise was stopped. Percent recovery metrics were calculated as the percent reduction of each hemodynamic variable from peak exercise to recovery, for example, (exercise‐recovery)/exercise × 100%. Figure 1 demonstrates the unique inter‐group comparisons (hemodynamic groups) of rest‐exercise‐recovery behavior of O_2_ pathway components.

In comparison to HFpEF and pre‐capillary PH, the NCD group reached higher peak V̇O_2_ and, as expected, had higher %V̇O_2_r (Figure 1, Table 1). Among metrics that define V̇O_2_, peripheral extraction recovered quickly to baseline (rest stage) in the NCD participants and was statistically different from disease groups (HFpEF, pre‐capillary PH). The percent heart rate recovery (%HRr) was only significantly different between the NCD and HFpEF groups, and the percent stroke volume recovery (%SVr) was similar in all hemodynamic groups.

### 3.4 Peripheral extraction recovery pattern – physiological correlate of peak V̇O_2_

Regression analyses are summarized in Table 2. On univariate linear regression to define correlates of peak exercise V̇O_2_, %SVr was not significant (*p* = 0.64), while %PEr (*r* ^2^ = 0.43, slope = 0.19, *p* < 0.001) and %HRr (*r* ^2^ = 0.25, slope = 0.23, *p* < 0.001) were significant.

**TABLE 2:** Correlates of peak V̇O_2_ with regression analyses.

Univariate linear regression					
Variable	*r* ^2^	Slope	Intercept	SE	*p*‐Value
Age	0.08	0.09	16.92	0.03	0.002
BMI	0.08	0.16	16.37	0.05	0.002
HTN	0.12	3.44	13.78	0.88	<0.001
Atrial fibrillation	0.08	2.79	12.24	0.88	0.002
H_2_FPEF score	0.29	1.14	15.9	0.17	<0.001
TAPSE	0.02	0.11	9.55	0.09	0.25
TAPSE/PASP	0.26	11.2	5.98	2.37	<0.001
Epicardial fat	0.02	0.24	12.61	0.17	0.17
LVMI	0.03	0.02	13.1	0.01	0.07
V̇O_2_% recovery	0.45	0.2	0.34	0.02	<0.001
PE % recovery	0.43	0.19	3.32	0.02	<0.001
Heart rate % recovery	0.25	0.23	7.1	0.04	<0.001
SV % recovery	0.05	0.03	11.16	0.06	0.64

Multivariate linear regression			
Adjusted *r* ^2^ = 0.698			
Variable	*β* coefficient	SE	*p*‐Value
Age	−0.13	0.06	0.03
BMI	−0.48	0.12	0.001
H_2_PEF score	0.72	0.44	0.12
TAPSE/PASP	9.98	3.41	0.01
Heart rate % recovery	0.08	0.07	0.28
PE % recovery	0.14	0.05	0.01

Abbreviations: BMI, body mass index; HTN, hypertension; LVMI, left ventricular mass index; PASP, pulmonary artery systolic pressure; PE, peripheral extraction; SE, standard error; SV, stroke volume; TAPSE, tricuspid annular pulmonary systolic excursion; V̇O_2_, oxygen consumption.

On multivariate regression analysis, all variables with *p* < 0.05 were included in the model (adjusted *r* ^2^ = 0.698, AIC = 149.6). Age, BMI, and TAPSE/PASP remained significant. However, with the goal of defining components of O_2_ pathway, %HRr did not remain significant (*p* = 0.28), and %PEr remained statistically significant (*p* = 0.01). This indicates that peripheral extraction recovery patterns have the strongest association with peak V̇O_2_, compared to recovery patterns of stroke volume and heart rate.

### 3.5 Prognostic value of peripheral extraction recovery

Survival analyses were performed based on the median value (> or ≤median value) of different components of O_2_ pathway (*n* = 94 due to lack of follow‐up of 26 participants). Based on median value, PEr ≤41% had the strongest correlation with poor survival from mortality and heart failure hospitalizations (HR 2.82, CI 1.38–5.74, *p* = 0.003, Figure 1). Using a similar method, V̇O_2_r ≤58% was also significant (HR 2.41, CI 1.20–4.86, *p* = 0.01).

## 4 DISCUSSION

In this study, we report a novel metric of percent peripheral extraction recovery (%PEr) among different PH phenotypes. %PEr was defined as the percent change of peripheral extraction from peak exercise to recovery. Among different components of the O_2_ pathway, %PEr had the strongest correlation with peak V̇O_2_. Moreover, participants with a median %PEr >41% were associated with positive clinical outcomes at 1 year. These findings indicate that peripheral extraction is the strongest correlate of exercise capacity and adverse clinical outcomes in a broad cohort of participants with suspected or diagnosed PH.

Poor O_2_ kinetics, including prolonged mean response time to peak V̇O_2_ (reflects inability to augment (Tang et al., 2017)) and prolonged V̇O_2_ recovery (reflects the metabolic debt from exercise) have also been associated with poor cardiac reserve and higher mortality (Bailey et al., 2018; Chatterjee et al., 2013). Systems‐level limitations from different components of the O_2_ pathway have also been reported (e.g., stroke volume and heart rate) (Houstis et al., 2018; Kitzman et al., 1991; Nayor et al., 2020). Impaired peripheral extraction and skeletal muscle disease have been shown to be a significant contributors to exercise intolerance, especially in HFpEF (Dhakal et al., 2015). In addition to the exercise behavior of these O_2_ pathway components, the recovery pattern of V̇O_2_ has also been associated with adverse clinical outcomes in both heart failure and non‐heart failure populations (Agostoni et al., 2024; Nanas et al., 2010). In our study, we quantitatively demonstrate that peripheral extraction recovery (%PEr, a novel metric related to the recovery phase) is the strongest physiologic contributor to the O_2_ pathway, with significant correlations with exercise capacity (peak V̇O_2_) and has prognostic significance (%PEr ≤41%). While our study highlights that peripheral extraction is a significant contributor to peak VO_2_, a recent study by Agostoni et al. delves deeper into changes in VO_2_ components after specific interventions in heart failure patients (exercise training, cardiac resynchronization therapy or percutaneous edge‐to‐edge mitral valve repair). Agostoni et al. demonstrated that improvements in peak V̇O_2_ after these interventions had a variable relationship with improvements in cardiac output or peripheral extraction. Hence, post‐intervention, blood flow was redistributed (centrally and peripherally) and contributed to V̇O_2_ improvements in a variable manner, highlighting the need to report both cardiac output and peripheral extraction improvements in studies involving clinical interventions (Agostoni et al., 2024).

Limitations include a single‐center, retrospective study. The current study does not account for the distribution of blood flow, which remains a major unsolved question and is beyond the scope of this study. Additionally, for O_2_ kinetics, a mono‐exponential model should ideally be applied, which assumes that V̇O_2_ and its components decay in an exponential manner post‐exercise; albeit it requires at least 2 data points in the recovery phase. In this study with a single recovery data point, we cannot fit a mono‐exponential model, which is a major limitation. Future studies are needed for validation and to further delineate these relationships in specific PH sub‐phenotypes and define the mechanistic underpinnings of poor skeletal muscle health in post‐exercise recovery.

## 5 CONCLUSION

Peripheral extraction recovery pattern is the most significant component of O_2_ pathway recovery post‐exercise. This novel metric correlates with exercise capacity (peak V̇O_2_) and %PEr >41% has prognostic value among patients with pulmonary hypertension and heart failure.

## FUNDING INFORMATION

National Center for Advancing Translational Sciences, Grant/Award Number: KL2TR002374–07; American Heart Association, Grant/Award Number: 23CDA1057697.

## CONFLICT OF INTEREST STATEMENT

None.

## ETHICS STATEMENT

The study received approval from the University of Wisconsin instituitional review board (IRB ID 2019‐0535) and informted consent was wavied due to retrospective nature of the study. The study adhered to the Declaration of Helsinki guidelines.

## TWEET

Breaking down the recovery of O_2_ pathway in HFpEF and Pre‐capillary PH: Peripheral extraction recovery emerges as a powerful correlate of exercise capacity, linking to survival and heart failure hospitalizations.
